# Effect of Translation-Enhancing Nascent SKIK Peptide on the Arrest Peptides Containing Consecutive Proline

**DOI:** 10.1021/acssynbio.4c00221

**Published:** 2024-11-22

**Authors:** Yuma Nishikawa, Riko Fujikawa, Hideo Nakano, Takashi Kanamori, Teruyo Ojima-Kato

**Affiliations:** † Laboratory of Molecular Biotechnology, Graduate School of Bioagricultural Sciences, Nagoya University, Furo-cho, Chikusa-ku, Nagoya 464-8601, Japan; ‡ GeneFrontier Corporation, 273-1 Kashiwa, Kashiwa, Chiba 277-0005, Japan

**Keywords:** polyproline, ribosome arrest peptides, SKIK, translation enhancement, EF-P

## Abstract

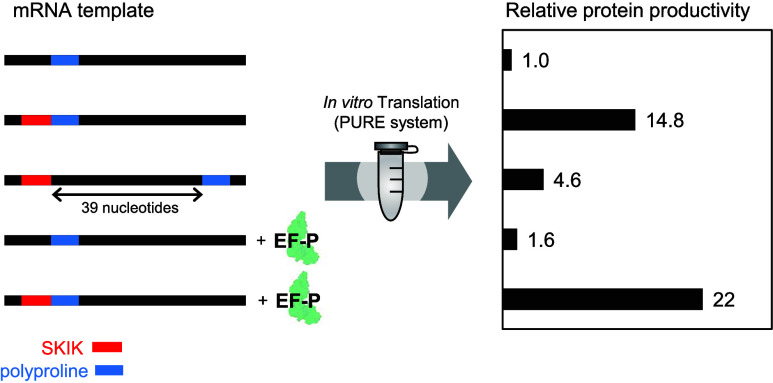

Ribosome arrest peptides (RAPs) such as the SecM arrest peptide (SecM AP: FSTPVWISQAQGIRAGP) and WPPP with consecutive Pro residues are known to induce translational stalling in *Escherichia coli*. We demonstrate that the translation-enhancing SKIK peptide tag, which consists of four amino acid residues Ser-Lys-Ile-Lys, effectively alleviates translational arrest caused by WPPP. Moreover, the proximity between SKIK and WPPP significantly influences the extent of this alleviation, observed in both PURE cell-free protein synthesis and in vivo protein production systems, resulting in a substantial increase in the yield of proteins containing such RAPs. Furthermore, we unveil that nascent SKIK peptide tag and translation elongation factor P (EF-P) alleviate ribosome stalling in consecutive-Pro-rich protein to synergistically promote translation. A kinetic analysis based on the generation of superfolder green fluorescent protein under in vitro translation reaction reveals that the ribosome turnover is enhanced by more than 10-fold when the SKIK peptide tag is positioned immediately upstream of the SecM AP sequence. Our findings provide valuable insights into optimizing protein production processes, which are essential for advancing synthetic biology applications.

## Introduction

Protein synthesis is a fundamental process in synthetic biology, and translation by ribosomes is an essential step in protein synthesis. Nonetheless, achieving high productivity for all proteins of interest remains a challenge, often leading to encounters with difficult-to-express proteins for reasons yet to be fully elucidated. Addressing this issue necessitates the consideration of various strategies. These include codon optimization,^1−6^ loosening the secondary structure of mRNA,^7−9^ and utilizing a combination of host–vector systems comprising ribosome-binding site (RBS), promoter, and terminator.^10−13^ Furthermore, the coexpression of molecular chaperons^14^ and the use of fusion protein tags (e.g., maltose-binding protein domain, glutathione S-transferase, small ubiquitin-related modifier) have proven beneficial.^15,16^ Optimizing culture conditions also play a pivotal role in enhancing productivity.^17−19^

Recently, some nascent polypeptides generated during the translation process have been reported as ribosome arrest peptides (RAPs), which cause translational stalling by interacting with internal components of the ribosome when they pass through the ribosome exit tunnel in the nascent state. RAP-mediated translation arrest is directed by the amino acid sequence of the nascent polypeptide chain instead of the mRNA nucleotide sequence. It is independent of codon usages or the secondary structure of mRNA.^21^

RAPs have been found in a variety of organisms, including SecM and the CmlA leader in *E. coli*,^20−23^ VemP in *Vibrio alginolyticus*,^24,25^ and MifM in *Bacillus subtilis*.^26^ Among them, SecM is the most studied, and the partial sequence F^150^XXXXWIXXXXGIRAGP^166^ (X is any amino acid) at the C-terminus is required and sufficient for causing ribosomal stalling,^27^ where new peptidyl bond formation is inhibited when the 166th Pro is about to be synthesized (P-site: SecM_1–165_-Gly-tRNA, A site: Pro-tRNA) at the peptidyltransferase center (PTC). Due to the structural rigidity and unique status as an *N*-alkylamino acid, Pro-tRNA at the A site slows down the peptide bond formation in the RAPs containing SecM and poly-Pro.^28^ As such, the WPPP (FXXYXIWPPP: X is any amino acid) sequence, artificially created RAP, efficiently induces ribosomal stalling due to its consecutive Pro residues.^29,30^ The universally conserved bacterial elongation factor called elongation factor P (EF-P, PDB 1UEB) is responsible for preventing the translational stalling caused by consecutive Pro in prokaryotic cells.^31−34^ The shape and size of EF-P are similar to tRNA, and it interacts with the ribosome via the E site in the PTC.^35−37^ However, Woolstenhulme et al. showed that EF-P had little or no effect on stalling by the full-length WPPP AP (FXXYXIWPPP) and RXPP motif.^30^

We have previously reported that the insertion of an “SKIK peptide tag”, composed of the four amino acids Ser-Lys-Ile-Lys at the N-terminus of difficult-to-express proteins, enhances protein production not only in both *E. coli* in vivo and in vitro systems but also in yeast.^38^ More recently, we revealed that changing nucleotide sequences corresponding to these four amino acids had no effect on protein production and that the nascent SKIK peptide generated during the translation process could enhance the translation process, but not transcription.^39^ Surprisingly, ribosomal stalling by RAPs such as SecM AP (FSTPVWISQAQGIRAGP) and chloramphenicol-induced CmlA leader (KNAD) was canceled by a nascent SKIK peptide generated immediately upstream of the RAPs. Moreover, the combined investigation of the SKIK peptide tag and SecM AP suggested that the SKIK peptide, even when positioned internally within the protein, could enhance translation. Additionally, it was suggested that the distance between SKIK and the AP motif might be crucial for SKIK to effectively alleviate translational stalling induced by the RAPs. However, it should be noted that in our previous study, the SKIK peptide tag showed no significant impact on translation when it was positioned immediately upstream of the WPPP AP (FQKYGIWPPP).^39^

Based on our recent findings, we hypothesized that ribosomal stalling induced by RAPs containing a poly-Pro motif could be alleviated by optimizing the distance between the nascent SKIK peptide and the poly-Pro motif. In this study, we investigated the positional effects of the SKIK peptide tag on ribosomal stalling caused by the poly-Pro AP motif in both *E. coli* in vitro and in vivo systems. Additionally, we discovered that EF-P and the SKIK peptide tag synergistically enhance translation, resulting in a significant increase in protein production. Translation promotion was observed only when the SKIK peptide was tagged and not by adding the synthesized free SKIK peptide to the in vitro translation system, emphasizing the importance of the nascent state of the SKIK peptide in counteracting arrest. Furthermore, we performed a kinetic analysis using an in vitro translation system to elucidate how the SKIK peptide tag influences translation.

## Results and Discussion

### Influences of the Distance between the SKIK and WPPP Arrest Peptides

**Figure 1:**
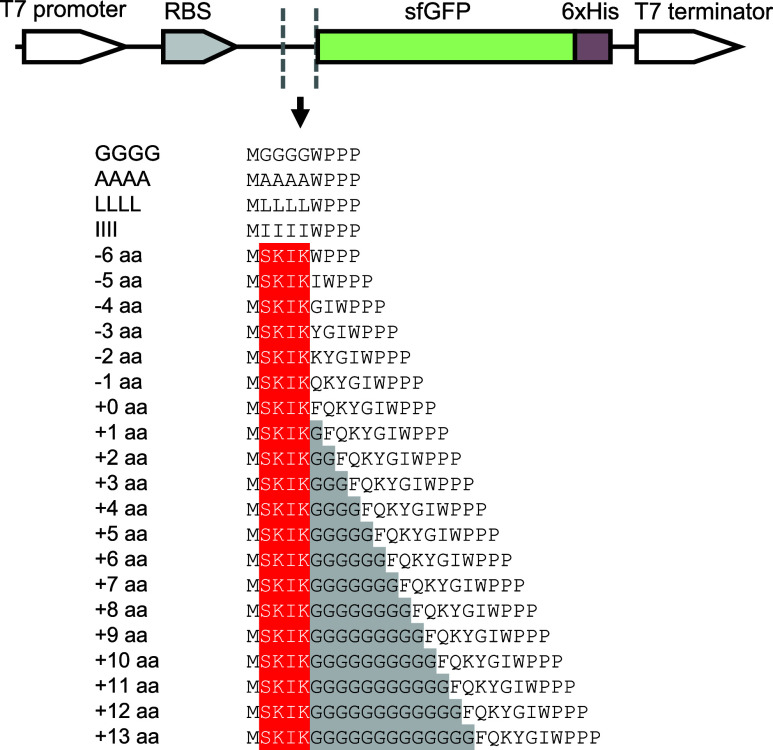
DNA constructs used to study the distance between the SKIK peptide tag and poly-Pro motif of WPPP. The amino acid sequences, including start M encoded immediately upstream of sfGFP, are shown. SKIK and inserted G are shown in red and gray, respectively.

**Figure 2:**
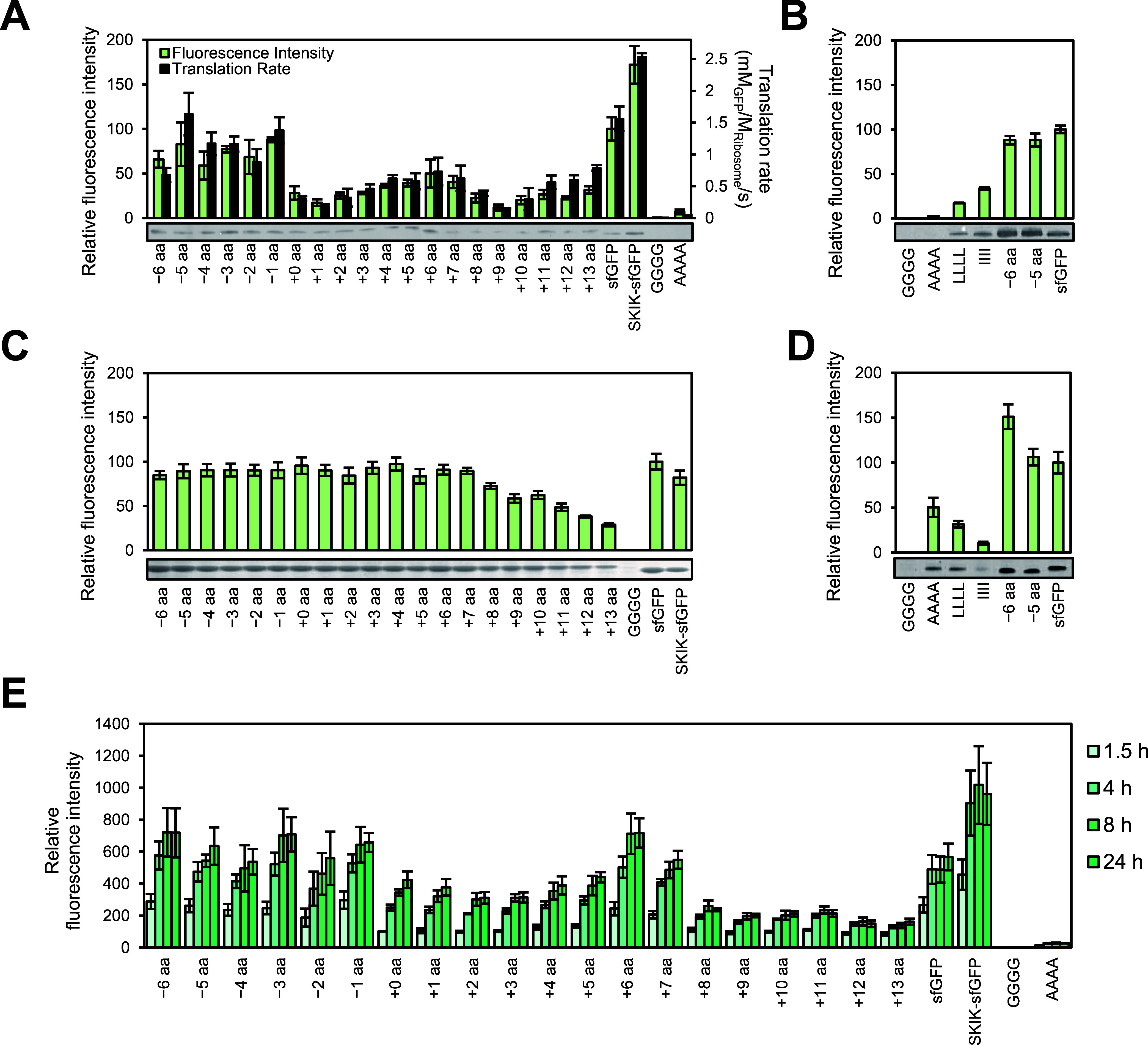
Influences of the distance between the SKIK peptide and the WPPP poly-Pro motif on protein production and translation rate. The sample names and sequences correspond to those of Figure 1. Fluorescence intensity in A, B, C, and D is shown as relative values, with the intensity of sfGFP regarded as 100. (A) Analysis of in vitro translation. Fluorescence intensity and Western blotting result of sfGFP detecting His tag, along with the translation rate (rate of generating sfGFP per ribosome per time) calculated from real-time monitoring of fluorescence intensity during in vitro translation. In vitro translation was carried out using mRNA templates for 90 min (*n* = 3, mRNA concentration is 500 ng/μL). (B) Effect of the peptide sequences other than SKIK (GGGG, AAAA, LLLL, and IIII) on in vitro translation. Fluorescence intensity of sfGFP and Western blot analysis detecting the His tag are shown. In vitro translation was carried out using mRNA templates for 90 min, which is the same as (A) (*n* = 3). (C) Analysis of the in vivo expression in *E. coli* BL21(DE3) cells. The fluorescence intensity of the produced sfGFP using the autoinduction system (*n* = 6) is shown, along with the corresponding sfGFP bands visualized by CBB staining on an SDS-PAGE gel. (D) Effect of the peptide sequences other than SKIK (GGGG, AAAA, LLLL, and IIII) on in vivo expression in *E. coli* BL21(DE3) cells. The fluorescence intensity of the produced sfGFP (*n* = 3) and the corresponding sfGFP bands visualized by CBB staining on SDS-PAGE are shown. (E) Time-dependent in vitro translation analysis. Fluorescence intensity during in vitro translation using mRNA templates (420 ng/μL, *n* = 3) was measured at each time point (1.5, 4, 8, and 24 h). The fluorescence intensity of all samples at each reaction time was normalized by regarding the intensity of +0 aa at 1.5 h as 100.

As described above, the ribosomal stalling by WPPP (FQKYGIWPPP), an artificial AP containing consecutive Pro, was not affected by the SKIK peptide tagging, while the ribosomal stalling by SecM AP was more effectively reduced when the SKIK peptide tag was positioned closer.^39^ We, therefore, created DNA constructs corresponding to the amino acid sequence depicted in Figure 1 to explore the hypothesis that an optimal position for the SKIK peptide tag exists concerning the poly-Pro motif. Here, the SKIK tag positioned immediately before the reported AP motif (FQKYGIWPPP) of WPPP was designated as the standard (+ 0 aa). Minus and plus numbers indicate the count of deleted amino acid residues from F in the WPPP AP motif and inserted G, respectively. To confirm that only the four amino acids “WPPP” function as an arrestor, a version with −6 aa (SKIKWPPP), where the SKIK moiety was substituted with GGGG (coding nucleotide: GGTGGCGGAGGG, GC contents = 83%), AAAA (coding nucleotide: GCAGCTGCCGCG, GC contents = 83%), LLLL (CTTCTCCTACTG, GC contents = 50%), or IIII (ATCATTATCATA, GC contents = 17%), was also constructed. In the results of in vitro translation using mRNA, the fluorescence intensity, reflecting the synthesized sfGFP quantity after 90 min, demonstrated a rising trend as the distance between SKIK and WPPP decreased (Figure 2A). Particularly noteworthy was the relative fluorescence intensity at −5 aa (more than 80), where SKIK and WPPP AP were brought closer, being approximately 3 times higher than at +0 aa (28), indicating the effective alleviation of ribosomal stalling induced by poly-Pro by the SKIK peptide tag. Western blotting analysis for His tag detection also revealed an increase in the full-length protein synthesis as the distance between SKIK and poly-Pro decreased. Moreover, insertions of G at +4–7 aa and +13 aa also exhibited approximately twice the fluorescence intensity of +0 aa, suggesting the possibility of multiple conditions where SKIK can effectively mitigate the ribosomal stalling caused by poly-Pro. It is worth noting that the relative fluorescence intensity for −6 aa (SKIKWPPP) was more than 50, whereas GGGG (sequence GGGGWPPP) and AAAA (AAAAWPPP) exhibited significantly lower intensities. Since the nucleotide sequences encoding these two control peptides were GC-rich, we also examined another control peptide encoded by lower GC content, namely, LLLL (sequence LLLLWPPP) and IIII (sequence IIIIWPPP), and found that these two showed less than half the fluorescence intensity of −6 aa (Figure 2B). This indicates that, at the least, the four amino acids WPPP alone can induce translation stalling at a certain degree. The strength of poly-Pro-induced translational stalling may be influenced by upstream sequences.^40,41^ Recent study by Kobo et al. showed that tetrapeptides other than SKIK could also alleviate SecM AP-induced translational stalling.^42^ Therefore, it is not surprising that IIII and LLLL apparently alleviate poly-Pro-induced stalling to some extent, although not as dramatic as SKIK. Since there are theoretically 160,000 types of tetrapeptides, it is unlikely that only SKIK can alleviate translation stalling. It is reasonable to assume that other peptides, to varying degrees, also influence translation. Nonetheless, to the best of our knowledge, this is the first report that SKIK has the potential to alleviate the translational arrest induced by poly-Pro, and its effectiveness seems to depend on the distance between SKIK and the poly-Pro motif.

The translation rate is defined as an active GFP synthesizing rate here, which is determined by monitoring the real-time synthesis of sfGFP from mRNA, correlating with the amount of the protein obtained after 90 min of reaction (Figure 2A). Hence, under the 90 min cell-free protein synthesis (CFPS) conditions in this experiment, it can be inferred that the final protein amount is indeed influenced by the translation rate. Nevertheless, as the distance increased beyond “+ 8 aa,” a declining trend in synthesis levels was observed. Upon comparison with the protein production levels of “GGGG,” a sequence excluding SKIK, it can be inferred that translational arrest was alleviated in all sequences containing SKIK, albeit with varying degrees.

The more prominent trend of translational arrest alleviation observed in vivo may be attributed to the fact that in vitro reactions were only 90 min, while, in vivo, we conducted longer expression by autoinduction system in which protein expression is induced during the late growth phase of *E. coli*, after the depletion of specific carbon sources (Figure 2C,D). Additionally, the presence of natural EF-P or unknown factors like the ribosomal quality control system in the living cells could contribute to this difference. In vitro, the reaction time was extended up to 24 h, but the sfGFP production still showed a correlation with the amount produced at 90 min (Figure 2E). This suggests that it may be challenging to accurately compare and analyze the differences in production between the in vivo and in vitro systems.

To clarify whether SKIK functions even when encoded internally within the gene, we conducted in vitro translation experiments using new constructs based on our previous findings, where internal SKIK or MSKIK alleviated SecM AP even more than 100 amino acids downstream from the start codon (Figure S1).^39^ In this study, the SecM sequence without the AP region (112 amino acid residues) was introduced. However, when SKIK was encoded internally, expression levels were significantly lower compared to N-terminal SKIK, and there was no difference in expression regardless of the presence or absence of internal SKIK. From these results, we concluded that SKIK promotes translation and alleviates downstream ribosomal stalling only when positioned at the N-terminus in cases where stalling is caused by consecutive Pro. In contrast, in the combination of SecM AP and SKIK, both the N-terminal and internal SKIK showed a stalling-alleviating effect in our previous study. The observed stalling-alleviating effect may depend on both the type of translational arrest and the surrounding sequences,^42^ likely because the mechanisms of translation arrest by SecM AP and consecutive Pro are different.

That said, to the best of our knowledge, there have been no reports on the function of the SKIK peptide to alleviate the ribosomal stalling by poly-Pro or on the importance of its positional relationship. Therefore, our findings will provide a critical and novel insight into the field of synthetic biology.

### Kinetic Analysis of the Protein Synthesis

**Figure 3:**
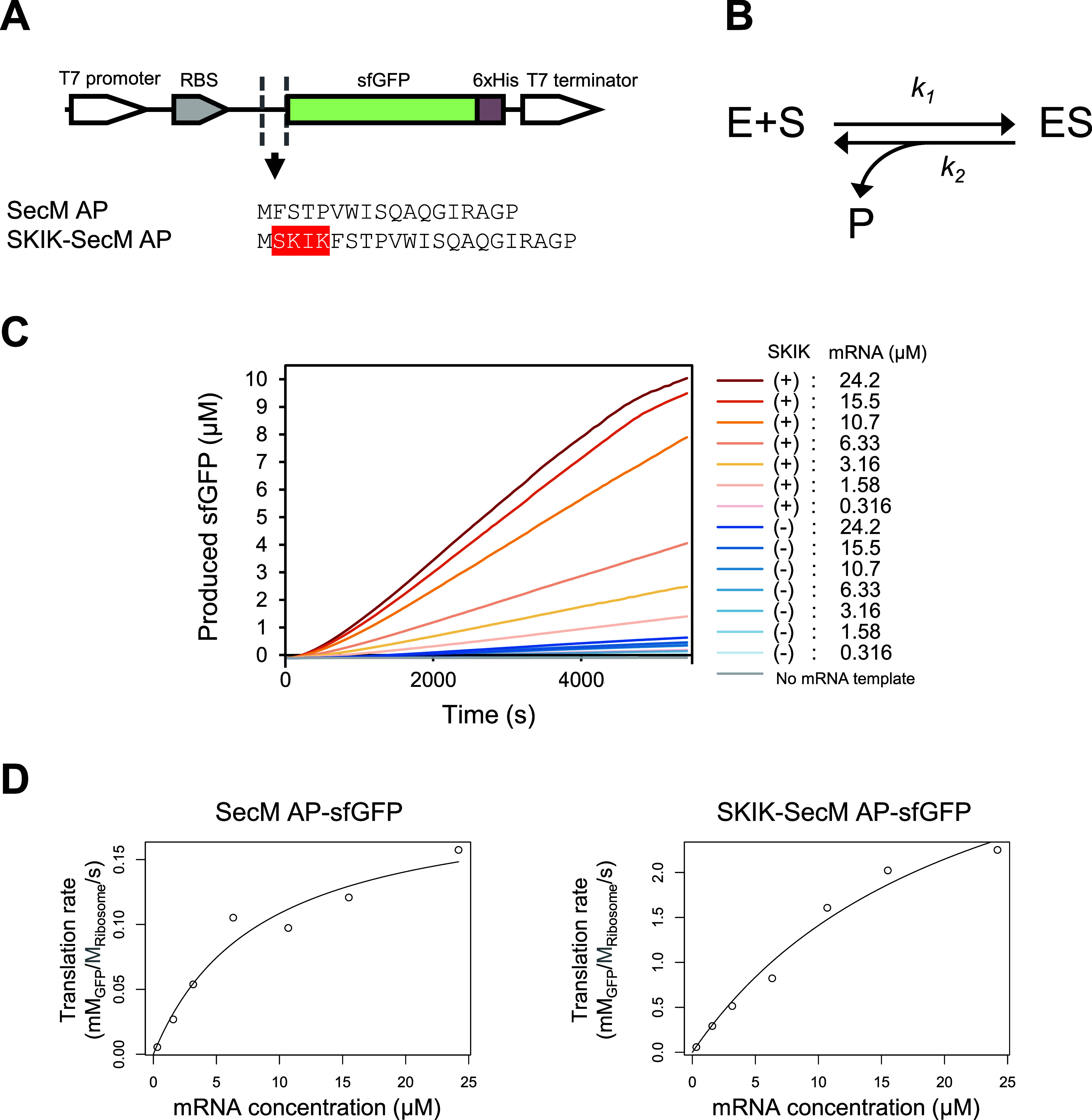
Kinetic analysis of translation by PURE cell-free protein synthesis. (A) mRNA constructs and amino acid sequences of SecM AP and SKIK-SecM AP. (B) Translation reaction model: E, S, and P represent the ribosome, mRNA, and sfGFP (product), respectively. *k*_1_ and *k*_2_ denote the rate constants for the reactions, indicated by the respective arrows. The ES complex encompasses all stages, from initiation to termination of translation. (C) Real-time fluorescence intensity measurements. Generation of sfGFP of various mRNA concentrations was monitored during a 90 min (5400 s) CFPS reaction. Plus and minus in the parentheses indicate the presence and absence of the SKIK peptide tag, respectively. (D) Nonlinear regression analysis of translation rate.

We then performed a kinetic analysis of translation to better understand the effect of the SKIK peptide tag on translation. Here, SecM AP-sfGFP with and without the SKIK peptide tag and simple translation reaction model was used because SKIK showed a significant effect in alleviating the ribosomal stalling caused by SecM AP (Figure 3A,B).^39^ For this analysis, we designate mRNA as “Substrate (S),″ sfGFP as “Product (P),″ and ribosome as “Enzyme (E)″ for convenience. We applied the following three assumptions to simplify the analysis: (1) there is no folding rate limitation for sfGFP, (2) there are sufficient elements necessary for translation, and no molecules inhibit the translation reaction, and (3) mRNA and ribosome do not dissociate until the translation of single protein molecule is completed.

The translation rate *v* was defined as follows.
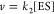
1

The following equation is given by the steady-state approximation.
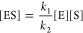
2

On the other hand, [E] can be expressed as follows:
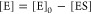
3

The following equation can be derived by substituting (3) into (2).
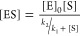
4

Based on the assumption that *k*_2_[E]_0_ = *V*_max_, substituting eq 1 into eq 4 results in a Michaelis–Menten-like equation as follows:
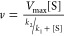
5where [S] = *k*_2_/*k*_1_ when the translation rate is 1/2 *V*_max_.

The translation rate was assessed by continuously monitoring the fluorescence intensity of sfGFP generated by CFPS using mRNA templates ranging from 0.316 to 24.2 μM. As shown in Figures 3C and S3, the rate of sfGFP production escalated proportionally with the quantity of the mRNA template utilized. Based on these measurements, the translation rate, which was defined as the rate of active GFP generation, was calculated (Table 1). It was observed that the translation rate increased by over 10-fold when the peptide was tagged by SKIK, regardless of mRNA concentrations, peaking at a maximum enhancement of 16.8-fold at 15.5 μM.

**Table 1:** Translation Rate

	*v* (mM_GFP_/M_Ribosome_/s)		
mRNA (μM)	no tag	SKIK	*v*_SKIK_/*v*_notag_
24.2	0.157	2.25	14.3
15.5	0.121	2.02	16.8
10.7	9.73 × 10^–2^	1.61	16.5
6.33	0.105	0.823	7.82
3.16	5.38 × 10^–2^	0.514	9.56
1.58	2.69 × 10^–2^	0.292	10.9
0.316	5.48 × 10^–3^	5.72 × 10^–2^	10.4
no template	9.52 × 10^–4^		

Utilizing the translation rates calculated for each mRNA concentration in Table 1, nonlinear regression analysis was conducted using the *R* package “renz″^43^ (Figure 3D). The regression curve aptly fits the experimental data, enabling the calculation of *V*_max_ and *k*_2_/*k*_1_ based on eq 5. As a result, the *V*_max_ values for the absence and presence of the SKIK peptide tag were determined to be 0.20 mM_GFP_/M_Ribosome_/s and 4.50 mM_GFP_/M_Ribosome_/s, respectively. The addition of the SKIK peptide tag led to a 22.5-fold increase in *V*_max_.

The calculated *k*_2_/*k*_1_ values were 8.45 μM without SKIK and 21.9 μM with SKIK, representing a 2.59-fold increase upon the addition of the SKIK peptide tag (Table 2). [E]_0_ = 1 μM and *V*_max_ = *k*_2_ [E]_0_ were used to calculate *k*_2_, and subsequently, *k*_1_ was calculated from the nonlinear regression analysis-derived *k*_2_/*k*_1_. As evident from this, the addition of the SKIK peptide tag led to values of *k*_1_ and *k*_2_ that were 8.65-fold and 22.5-fold higher, respectively.

**Table 2:** Parameters on Translation Calculated Based on Real-Time Translation Monitoring

	*V*_max_ (mM_GFP_·M_Ribosome_^–1^·s^–1^)	*k*_2_/*k*_1_ (μM)	*k*_1_	*k*_2_
no tag	0.20	8.45	2.37 × 10^7^	0.20 × 10^3^
SKIK	4.50	21.9	20.5 × 10^7^	4.50 × 10^3^
SKIK/no tag (-fold)	22.5	2.59	8.65	22.5

Since the RBS is the same in the two examined mRNA sequences, it seems unlikely that the affinity between the ribosome and mRNA changes significantly. Therefore, the increase in *k*_1_ resulting from the addition of the SKIK peptide tag suggests an augmentation in the pool of ribosomes capable of initiating translation. Similarly, the increase in *k*_2_ implies an acceleration in the rate of translation from the initiation to termination. These kinetic analyses indicate that the addition of the SKIK peptide tag could enhance the ribosomal turnover rate.

Regarding studies on the kinetic analysis of translation, previous analyses have utilized the incorporation of radiolabeled amino acids into synthesized proteins;^44^ more recently, elongation rates per codon have been calculated based on ribosome density observed on mRNA through ribosome profiling.^45,46^ To the best of our knowledge, our analysis, based on the production of sfGFP as an indicator, is the first attempt in this direction. The elongation rate per codon is commonly used as an indicator of translation efficiency, with reported values ranging from 10 to 20 codons/s in *E. coli* in vivo^47^ to 10 codons/s in vitro,^44^ 4.2 codons/s in *S. cerevisiae*, and 5.2 codons/s in mouse stem cells.^45^

In this study, the SKIK-SecM AP-sfGFP gene used consists of 267 codons. The apparent elongation rate per codon calculated from the estimated *V*_max_ of SKIK-SecM AP-sfGFP was 1.2 codons/s. Although this value is lower than those reported in previous studies, considering that ribosomal stalling by SecM is not completely alleviated by the SKIK peptide tag,^39^ and our reaction model disregards the translation initiation and termination stages, which require time, the calculated apparent elongation rate appears reasonable. Although it should be noted that the use of this model might be limited, we concluded that the simple translation rate analysis method proposed in this study worked well for our purpose.

### Effect of EF-P and the SKIK Peptide

**Figure 4:**
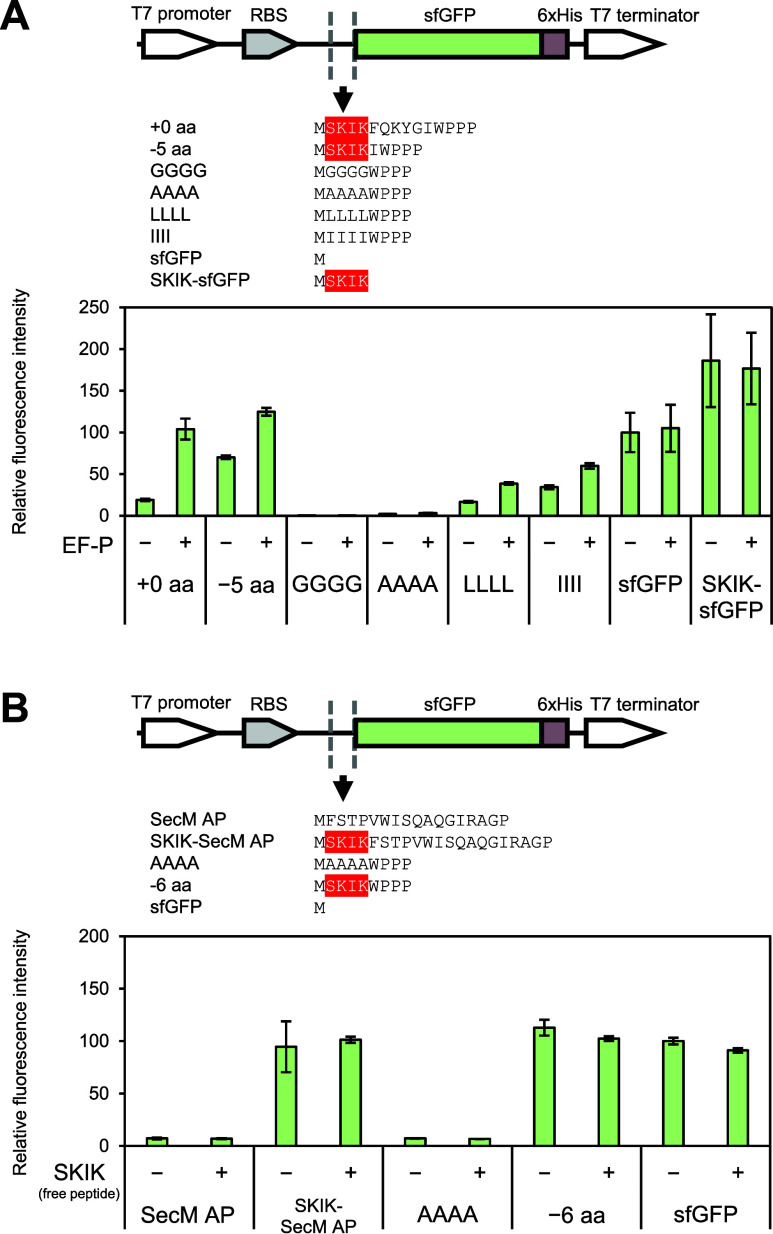
Effect of EF-P and the free SKIK peptide on protein production in CFPS. (A) Relative fluorescence intensity of sfGFP by in vitro translation using mRNA (420 ng/μL) after 90 min reaction (*n* = 3). The plus/minus (±) symbol indicates the presence/absence of EF-P (1 μM). The fluorescence intensity of sfGFP without EF-P was regarded as 100. (B) Relative fluorescence intensity of sfGFP by in vitro translation using mRNA after 90 min reaction. The plus/minus (±) symbol indicates the presence/absence of synthesized free SKIK peptide (100 μM). The fluorescence intensity of sfGFP without free SKIK peptide was regarded as 100.

EF-P is a translation factor reported to promote the synthesis of the poly-Pro motif. Therefore, we investigated how the SKIK peptide tag, capable of relieving ribosomal stalling induced by poly-Pro, and EF-P would affect the translation of sequences containing poly-Pro. We examined using eight sequences including −5 aa, +0 aa, GGGG, AAAA, LLLL, IIII, original sfGFP, and SKIK-tagged sfGFP shown in Figure 4A. Among these, the ribosomal stalling was most effectively alleviated by the SKIK peptide tag alone in −5 aa (Figure 2A). As a result of EF-P addition at a final concentration of 1 μM, the protein production was increased to 1.5-fold and 4.4-fold in −5 aa and +0 aa, respectively, compared with no additive. In particular, the protein production was highest for −5 aa with EF-P, indicating a greater synergistic effect by the SKIK peptide tag and EF-P (Figure 4A). On the contrary, sfGFP and SKIK-sfGFP, which do not contain the poly-Pro motif, were not affected by the presence or absence of EF-P in terms of production levels. It suggests the possibility that EF-P and the SKIK peptide tag individually promote translation in this case. By contrast, GGGG and AAAA, which do not contain SKIK, did not show any change in protein levels with the addition of EF-P. Translation levels for LLLL and IIII also increased in the presence of EF-P but with less effect than for SKIK.

To confirm that the nascent state of the SKIK peptide is essential for promoting the translation or stalling-alleviating effect, CFPS of five mRNA templates was conducted with the addition of the chemically synthesized free SKIK peptides at 100 μM, which was 100 times higher than that of the ribosome in the CFPS reaction. As expected, no influence was observed (Figure 4B). Huter et al. demonstrated that EF-P recognizes the P-site tRNA and the E site mRNA codon, stabilizing the conformation of the P-site tRNA and thereby promoting peptide bond formation.^48^ Taken together with the results of the addition of free SKIK peptide and our previous study,^39^ it is likely that the nascent state of the SKIK peptide tag is crucial for translation promotion. Therefore, we expect that the mechanism of action of the externally functioning EF-P and SKIK peptide tags at the nascent state may be different. Since the mechanism of translation enhancement by the SKIK peptide tag has not yet been elucidated, we believe that further structural biological analyses, such as investigating the interactions between the ribosome and nascent peptides, will be necessary.

## Conclusions

In this study, we revealed that the translation-enhancing SKIK peptide tag effectively alleviates ribosomal stalling induced by the WPPP when the distance between them is closer in both the PURE cell-free protein synthesis and in vivo protein production systems when SKIK was encoded at the N-terminus, resulting in a significant increment of production of proteins containing such poly-Pro motif. A kinetic analysis based on the simple translation model showed that the ribosome turnover is enhanced by more than 10-fold when the SKIK peptide tag is present upstream of SecM AP. Furthermore, we found that the use of both the SKIK peptide tag and EF-P synergistically enhanced translation for proteins containing poly-Pro. The findings of our study and simple strategy have practical significance in enhancing protein production and contribute to the advancement of synthetic biology.

The translation rate *v* is defined as the rate of active GFP generation and calculated from the plot shown in Figure 3D, and the ratio of *v* with and without SKIK is presented as *v*_SKIK_/*v*_No tag_. No tag or SKIK means SecM AP-sfGFP and SKIK-SecM AP-sfGFP, respectively.

*V*_max_ and *k*_2_/*k*_1_ were calculated using nonlinear regression analysis with the renz software, as shown in Figure 3D. The rate constants *k*_1_ and *k*_2_ were estimated from the values of *V*_max_ and *k*_2_/*k*_1_ obtained through the nonlinear regression analysis. The units of *k*_1_ and *k*_2_ are M_GFP_·M_Ribosome_^–2^·s^–1^·M_mRNA_^–1^ and M_GFP_·M_Ribosome_^–2^·s^–1^, respectively. M_Ribosome_ and M_mRNA_ represent the concentrations of ribosome and mRNA in mol/L, respectively.

## Methods

### DNA Primer List

DNA primers used in this research are summarized in Supporting Information Table S1.

### Plasmid Construction

All plasmids were prepared by whole plasmid PCR using our previously constructed pET22b-SKIK-WPPP-sfGFP-His as the template and DNA primers listed in Supporting Information Table S1. *Dpn*I-treated PCR products were purified with a silica column (FastGene Gel/PCR Extraction Kit, Nippon Genetics Co., Ltd., Tokyo, Japan). Then, the HiFi assembly reaction (New England Biolabs, Ipswich, MA) was carried out to connect each end of linear DNA products. The plasmids were prepared using a plasmid mini-prep kit (FastGene Plasmid Mini Kit, Nippon Genetics Co., Ltd.) from overnight cultured *E. coli* DH5alpha transformants. The sequences of all plasmids confirmed by Sangar sequencing are available in the Supporting Information.

### Cell-Free Protein Synthesis (CFPS)

DNA fragments containing T7 promoter and terminator used for CFPS were prepared by PCR with the primer pair F1 and R1, constructed plasmids as the template, and Tks Gflex DNA polymerase (98 °C 1 min; (98 °C 10 s; 50 °C 5 s; 68 °C 30 s) × 30 cycles; 68 °C 1 min; 12 °C ∞, Takara, Kusatsu, Japan). Amplified DNA was purified using a silica column and used for in vitro mRNA synthesis. For in vitro mRNA synthesis, the T7 RiboMAX Large-Scale RNA Production System (Promega, Madison, WI) was used, and the synthesized mRNAs were purified with NucleoSpin RNA Plus (Takara). The concentration of mRNA was measured by NanoDrop One (Thermo Fisher Scientific, MA), and the molecular weight was calculated using RNA Molecular Weight Calculator on AAT Bioquest (https://www.aatbio.com).

CFPS reactions were carried out with PURE*frex* 2.1 (GeneFrontier Corporation, Kashiwa, Japan) under the following conditions: Solution I, 4 μL; Solution II, 0.5 μL; Solution III (20 μM ribosome), 0.5 μL; 10 mM cysteine, 0.5 μL; RNase Inhibitor (TOYOBO, Osaka, Japan), 1 μL; final reaction volume, 10 μL; incubation at 37°C for 90 min. The final concentration of the template mRNA depends on the examination. An elongation factor EF-P (GeneFrontier Corporation) and the synthesized SKIK peptide dissolved in nuclease-free water (94.6% purity, GenScript, Tokyo) were added to achieve a final concentration of 1 and 100 μM, respectively, as needed.

### In Vivo Expression

Terrific broth (Bacto tryptone, 12 g; yeast extract, 24 g; KH_2_PO_4_, 6.8 g; Na_2_PO_4_·12H_2_O, 7.1 g; MgSO_4_, 0.15 g; (NH_4_)_2_SO_4_, 2.58 g; 10%(w/v) glucose, 5 mL; and 8%(w/v) lactose; 25 mL, per 1 L, adjusted pH 7.4) was used as the autoinduction medium. The single colonies of ECOS *E. coli* BL21(DE3) (Nippon Gene, Tokyo, Japan) transformed with each plasmid were inoculated to 4 mL of LB liquid and grown with shaking at 37°C for 19 h. Then, the aliquots (50 μL) were transferred to 1 mL of terrific broth and cultured at 30 °C until the OD600 reached 2.3–6.9. All media were supplemented with 100 μg/mL of ampicillin. After collecting the bacterial cells by centrifugation and washing with phosphate buffer saline buffer (PBS, 137 mM NaCl, 2.7 mM KCl, 10 mM Na_2_HPO_4_, 1.8 mM KH_2_PO_4_; pH 7.4), the OD600 values of each sample were adjusted to the same. Protein was extracted from the cells using *E.coli*/Yeast Protein Extraction Buffer (Tokyo Chemical Industry Co., Ltd., Tokyo, Japan), and the lysate fraction was used for analysis.

### Preparation of Standard sfGFP

ECOS *E. coli* BL21(DE3) was transformed with pET22b-sfGFP-His and cultured in 100 mL of LB medium at 37 °C with shaking until the OD600 value reached 0.4–0.6. IPTG was then added at a final concentration of 0.1 mM and cultured at 37 °C for another 5 h. Bacterial cells were collected by centrifugation (4 °C, 9000 rpm, 10 min), resuspended in 10 mL of PBS, and then the bacteria were crushed by sonication (30 min). The supernatant of the lysate was applied to a Ni-NTA agarose HP column (Fujifilm Wako Pure Chemical Corporation, Osaka, Japan) equilibrated with Binding buffer (20 mM sodium phosphate buffer, 0.5 M NaCl, 20 mM imidazole, pH 8.0), and the column was washed three times with 6 mL of binding buffer and eluted six times with 1 mL of the elution buffer (20 mM sodium phosphate buffer, 0.5 M NaCl, 500 mM imidazole, pH 8.0). The eluate was added to the dialysis membrane and dialyzed for a total of 24 h (buffer was changed twice during the dialysis). The concentration of purified sfGFP was calculated from the A280 nm value (A.U.) measured by NanoDrop One. Molecular weight (27788.3 Da) and molar extinction coefficient (19,035 M^–1^·cm^–1^) of sfGFP were calculated using a ProtParam tool on Expasy (https://www.expasy.org).^49^

### Fluorescence Measurement

Each CFPS reaction solution was diluted 25-fold with water and dispensed 50 μL per well into Flat Bottom Microfluor Plates Black (Thermo Fisher Scientific, MA), and the fluorescence intensity of sfGFP was measured by a microplate reader (Infinite 200 PRO, TECAN, ZH, Switzerland) at an excitation wavelength of 485 nm (bandwidth 9 nm)/emission wavelength of 535 nm (bandwidth 20 nm).

### Real-Time Monitoring of the Translation in CFPS

The reaction was performed using a real-time PCR system (StepOnePlus Real-Time PCR System, Life Technologies, CA) by incubating at 37 °C, and the fluorescence intensity was measured every minute after 30 s. Fluorescence intensity per sfGFP molecule was calculated using purified sfGFP (14.6, 1.46, 0.146, 0.0146, and 0.00146 μM) as the standard. The translation rate, defined as the generated sfGFP per ribosome per time, was calculated from the rate of the fluorescence increase, where the slope was the greatest and linear for all samples.

### Western Blotting

The samples (1 μL) were subjected to SDS-PAGE and transferred to a nitrocellulose membrane (Bio-Rad Laboratories, CA). The membrane was blocked using 3% skim milk in PBST (137 mM NaCl, 2.7 mM KCl, 10 mM Na_2_HPO_4_, 1.8 mM KH_2_PO_4_, 0.05% Tween-20; pH 7.4) for 1 h at room temperature. After washing three times with PBST, the membrane was incubated with Anti-His-tag mAb-HRP-DirectT (MBL, Tokyo, Japan) at 1:5000 dilution with Can Get Signal Solution 2 (Toyobo, Osaka, Japan) for 1 h at room temperature. After being washed with PBST three times, the blots were visualized with the TMB solution (Nacalai Tesque, Kyoto, Japan).

## Supporting Information Available

DNA sequences for plasmid with GenBank format and additional experimental data (PDF)
Sequences of all plasmids confirmed by Sangar sequencing (ZIP)

## Abbreviations

RAP: ribosomal arrest peptide
RBS: ribosome-binding site
PTC: peptidyl transferase center
CFPS: cell-free protein synthesis
sfGFP: superfolder green fluorescent protein
