# Supplementary material for: Effect of Translation-Enhancing Nascent SKIK Peptide on the Arrest Peptides Containing Consecutive Proline
Source: ACS Synth Biol. 2024 Nov 22;13(12):3908–16. doi: 10.1021/acssynbio.4c00221 (PMC11669330; doi:10.1021/acssynbio.4c00221)
Supplement: Supplementary file 1 — sb4c00221_si_001.pdf [file sb4c00221_si_001.pdf]

Supporting Information

Effect of translation enhancing nascent SKIK peptide on the arrest peptides containing consecutive proline

Yuma Nishikawa[1], Riko Fujikawa[1], Hideo Nakano[1], Takashi Kanamori[2], Teruyo Ojima-Kato[1]*

[1] Laboratory of Molecular Biotechnology, Graduate School of Bioagricultural Sciences, Nagoya University, Furo-cho, Chikusa-ku, Nagoya 464-8601, Japan
[2] GeneFrontier Corporation, 273-1 Kashiwa, Kashiwa, Chiba 277-0005, Japan

*, the person to whom correspondence
https://orcid.org/0000-0001-5290-5176; E-mail: teruyo@agr.nagoya-u.ac.jp

Table S1. DNA primers used in this study.

Name	sequence (5′→3′)
G1_F_v2	AAATAAAAGGTTTCCAGAAGTACGGGATTTGGCC
G1_R_v2	TCTGGAAACCTTTTATTTTAGACATATGTATATCTCCTTCTTAAAG
G2_F	AAATAAAAGGTGGATTCCAGAAGTACGGGATTTGGCC
G2_R	CTTCTGGAATCCACCTTTTATTTTAGACATATGTATATCTCCTTCTTAAAG
G3_F	GGTGGAGGTTTCCAGAAGTACGGGATTTGGCC
G3_R_v2	CTTCTGGAAACCTCCACCTTTTATTTTAGACATATGTATATCTCCTTCTTAAAG
G4_F	ATAAAAGGTGGAGGTGGCTTCCAGAAGTACGGGATTTGG
G4_R	GCCACCTCCACCTTTTATTTTAGACATATGTATATCTCC
G5_F	GGTGGAGGTGGCGGATTCCAGAAGTACGGGATTTGGCC
G5_R	GAATCCGCCACCTCCACCTTTTATTTTAGACATATGTATATCTCCTTCTTAAAG
G6_F	GGTGGAGGTGGCGGAGGTTTCCAGAAGTACGGGATTTGGCC
G7_F	GGTGGAGGTGGCGGAGGTGGCTTCCAGAAGTACGGGATTTGGCC
G7<_R	ACCTCCGCCACCTCCACCTTTTATTTTAGACATATGTATATCTCCTTCTTAAAG
G8_F	GGTGGAGGTGGCGGAGGTTTCCAGAAGTACGGGATTTGGCC
G9_F	GGTGGAGGTGGCGGAGGTGGCTTCCAGAAGTACGGGATTTGGCC
G10_F	GGTGGAGGTGGCGGAGGTGGCGGTGGAGGATTCCAGAAGTACGGGATTTGGCC
G11_F	GGTGGAGGTGGCGGAGGTGGCTTCCAGAAGTACGGGATTTGGCC
G12_F	GGTGGAGGTGGCGGAGGTGGCGGTGGAGGAGGTGGTTTCCAGAAGTACGGGATTTGGCC
G13_F	GGTGGAGGTGGCGGAGGTGGCGGTGGAGGAGGTGGTGGATTCCAGAAGTACGGGATTTGGCC
F_Fw	AAAATAAAACAGAAGTACGGGATTTGGCCGC
FQ_Fw	AAAATAAAAAAGTACGGGATTTGGCCGCCC
FQK_Fw	AAAATAAAATACGGGATTTGGCCGCCCCC
FQKY_Fw	AAAATAAAAGGGATTTGGCCGCCCCCTG
FQKYG_Fw	AAAATAAAAATTTGGCCGCCCCCTGCAAG
FQKYGI_Fw	AAAATAAAATGGCCGCCCCCTGCAAGTAAA
F_Rv	GTACTTCTGTTTTATTTTAGACATATGTATATCTCCTTCTTAAAG
FQ_Rv	CCCGTACTTTTTTATTTTAGACATATGTATATCTCCTTCTTAAAG
FQK_Rv	AATCCCGTATTTTATTTTAGACATATGTATATCTCCTTCTTAAAG
FQKY_Rv	CCAAATCCCTTTTATTTTAGACATATGTATATCTCCTTCTTAAAG
FQKYG_Rv	CGGCCAAATTTTTATTTTAGACATATGTATATCTCCTTCTTAAAG
FQKYGI_Rv	GGGCGGCCATTTTATTTTAGACATATGTATATCTCCTTCTTAAAG
G_replce_F	ATGGGTGGCGGAGGGTGGCCGCCCCCTGCAAGTAAAG
G_replce_Rv	CCACCCTCCGCCACCCATATGTATATCTCCTTCTTAAAGTTAAACAAAATTATTTC

A_replace_F	ATGGCAGCTGCCGCGTGGCCGCCCCCTGCAAGTAAAG
A_replace_R	CCACGCGGCAGCTGCCATATGTATATCTCCTTCTTAAAGTTAAACAAAATTATTTC
I_replace_F	ATGATCATTATCATATGGCCGCCCCCTGCAAGTAAAG
I_replace_R	CCATATGATAATGATCATATGTATATCTCCTTCTTAAAGTTAAACAAAATTATTTC
L_replace_F	ATGCTTCTCCTACTGTGGCCGCCCCCTGCAAGTAAAG
L_replace_R	CCACAGTAGGAGAAGCATATGTATATCTCCTTCTTAAAGTTAAACAAAATTATTTC
secM_insert_Fw	GAAGGAGATATACATATGGCC
secM_insert_Rv	CCAAATTTTTATTTTAGATTTTGCTTGTGGGGTAAAATG
SKIK_del_F	CAAGCAAAAATTTGGCCGCCCCCTGCAAG
SKIK_del_R	GCGGCCAAATTTTTGCTTGTGGGGTAAAATGCGC
F1	ATCTCGATCCCGCGAAATTAATACG
R1	TCCGGATATAGTTCCTCCTTTCAG

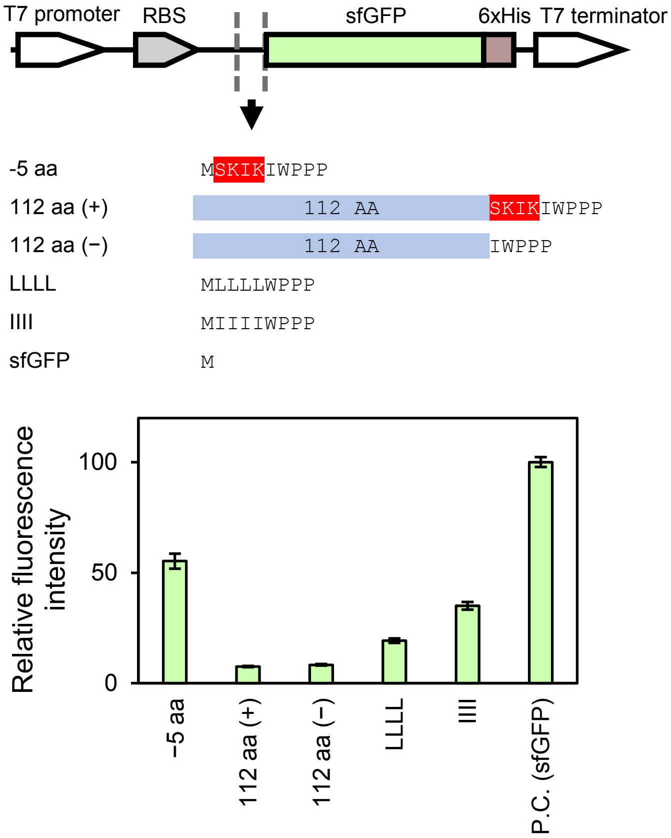

Figure S1. Comparison of the effect of N-terminal and internal SKIK on consecutive Pro.

The relative fluorescence intensity of in vitro translation using mRNA (420 ng/μL) for 90 minutes is shown (n=3). The region "112 aa" corresponds to the SecM sequence without AP region:

AEPNAPAKATTRNHEPSAKVNFGQLALLEANTRRPNSNYSVDYWHQHAIRTVIRHLSFAM
APQTLPVAEESLPLQAQHLALLDTLSALLTQEGTPSEKGYRIDYAHFTPQAK.

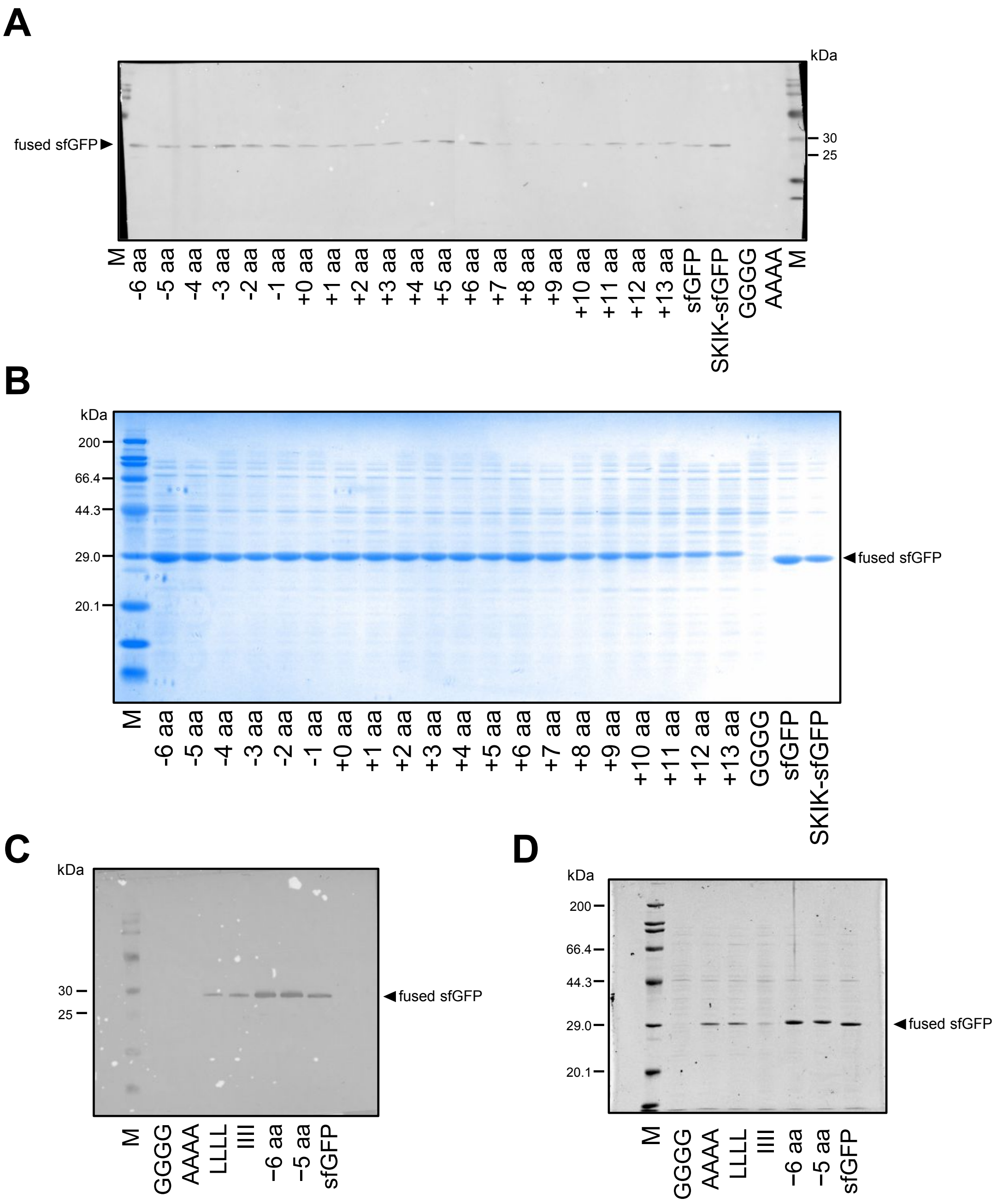

Figure S2. SDS-PAGE analysis of in vitro and in vivo protein production.

The full images of Western blotting and CBB staining in Figure 2 in the main manuscript are shown. The markers used were Protein MultiColor Stable II (BioDynamics Laboratory, Japan, Tokyo) and Protein Molecular Weight Marker (Broad) (Takara) in Western blotting and CBB staining, respectively.

A) Western Blotting in Figure 2A.

B) CBB staining result in Figure 2C.

C) Western Blotting in Figure 2B.

D) CBB staining result in Figure 2D.

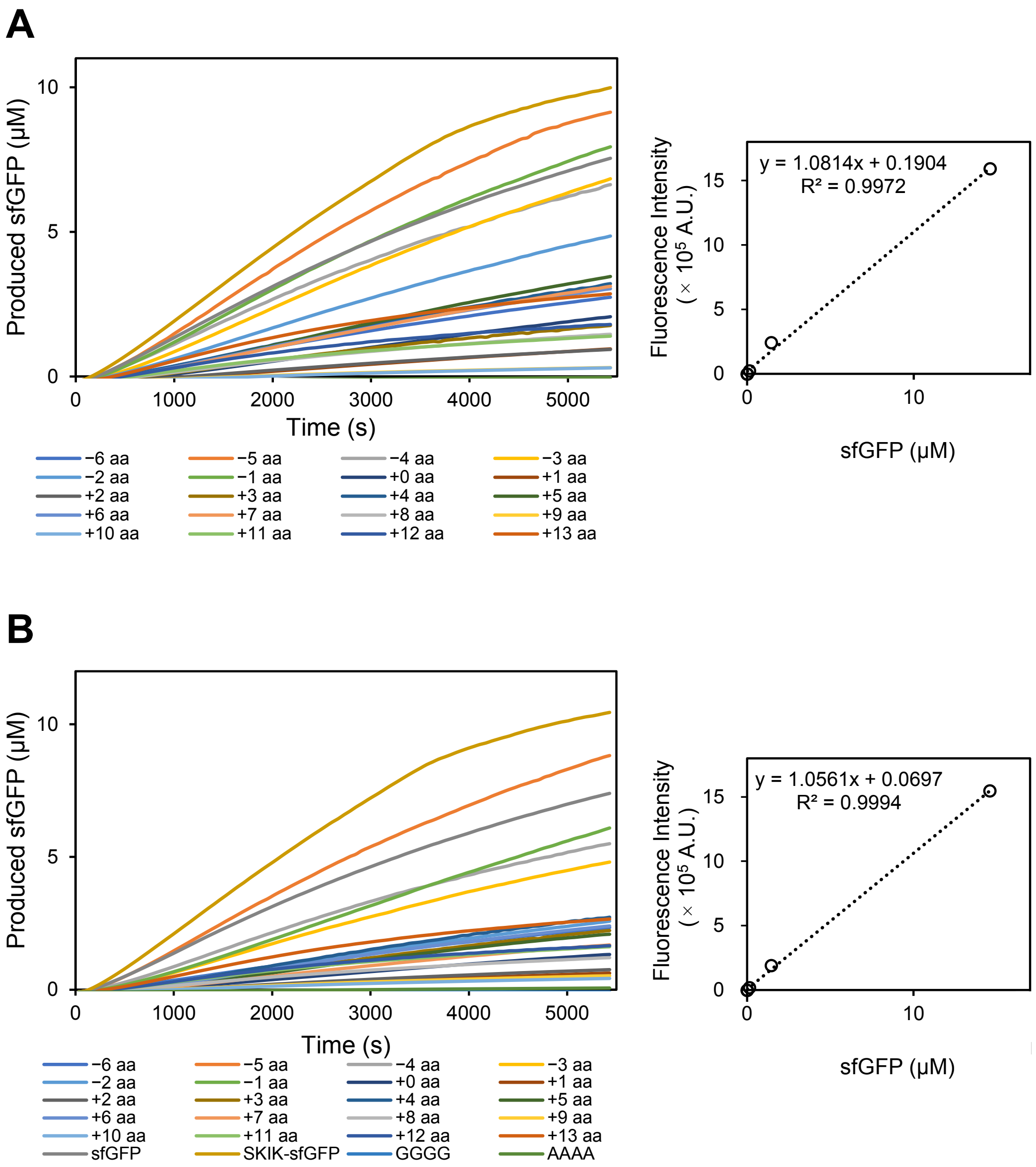

Figure S3. Real-time monitoring of the produced sfGFP in an in vitro translation. The sample names and sequences are corresponding to those of Figure 1. Fluorescence intensity of the produced sfGFP from mRNA was monitored during 90min (5,400 s) CFPS reaction to evaluate the influence of the distance between the SKIK peptide tag and WPPP poly-proline motif. Two independent monitoring results (left) and standard curves of the purified sfGFP (right) are shown as A and B. Translation rate in Figure 2B was calculated based on the rate of increase in sfGFP in the 450–990 s.
